# Patellar cartilage thickness relates to knee external rotation during squatting in individuals with and without patellofemoral pain—a pilot study

**DOI:** 10.3389/fspor.2025.1575115

**Published:** 2025-05-08

**Authors:** Hiraku Nagahori, Kai-Yu Ho

**Affiliations:** ^1^ Department of Physical Therapy, University of Nevada, Las Vegas, Las Vegas, NV, United States; ^2^ Division of Biokinesiology and Physical Therapy, University of Southern California, Los Angeles, CA, United States

**Keywords:** tibial rotation, patellofemoral pain, anterior knee pain, patellar cartilage, cartilage morphology, knee rotation

## Abstract

The relationship between patellofemoral cartilage morphology and knee external rotation (KER), one of the possible factors increasing patellar cartilage stress, has been rarely explored in individuals with and without patellofemoral pain (PFP). Ten individuals with PFP and 10 pain-free controls, matched for age, weight, height, and activity level, participated. Patellar cartilage morphology was assessed using 3-Tesla magnetic resonance imaging. Lower extremity kinematics during bilateral squatting at 45° of knee flexion were captured using a 3-dimensional motion capture system. Pearson and Spearman correlation coefficients were used to assess the associations between cartilage thickness (medial, lateral, and total) and peak KER, along with other peak joint angles across the three planes. Across all participants, there were significantly moderate correlations between medial cartilage thickness and KER (*r* = −0.48, *p* = 0.03), and total cartilage thickness and KER (*r* = −0.47, *p* = 0.35). In the PFP group, there was a significantly large correlation between medial cartilage thickness and KER (*r* = −0.66, *p* = 0.03). In the control group, there was a significant very large correlation between lateral cartilage thickness and KER (*r* = −0.79, *p* = 0.01) and a significant very large correlation between total cartilage thickness and KER (*r* = −0.75, *p* = 0.01). The findings suggest that thinner patellar cartilage is associated with increased KER during bilateral squatting in persons with and without PFP. Since our study focused on a double-limb activity, which may require less KER, future research should examine its impact on cartilage morphology during single-limb activities.

## 1 Introduction

Patellofemoral pain (PFP) refers to pain localized around the peripatellar and/or retropatellar regions, commonly occurring during activities that involve knee flexion under weight bearing, such as running and squatting (1, 2). The causes of PFP include irritation of the innervated structures of the patellofemoral joint (PFJ), such as the subchondral bone, often due to increased pressure on the cartilage of the PFJ (3–5). Repeated excessive loading of the PFJ during weight-bearing activities can lead to cartilage thinning and altered composition (6). Farrokhi et al. (6) have shown that individuals with PFP exhibit thinner patellar cartilage compared to pain-free controls. Thinner PFJ cartilage can increase stress on the cartilage (7), as it reduces the cartilage's ability to absorb and distribute force during weight-bearing. Additionally, a computational modeling study found a significant negative association between patellar bone strain and cartilage thickness, suggesting that thinner patellar cartilage may contribute to bone injuries and pain in individuals with PFP (8).

It is important to note that faulty lower extremity kinematics can contribute to increased stress on the PFJ. Specifically, in addition to hip adduction and internal rotation (9), knee flexion (10) and abduction (9), knee external rotation (KER) is reported as a contributing factor leading to elevated PFJ stress in a cadaveric study (11). It is believed that external rotation of the tibia shifts the patellar contact region laterally and increases the contact pressure on the lateral aspect of the patella (12). Specifically, KER during activities causes the tibial tuberosity to move laterally, which in turn pulls the patella laterally (13). This movement results in a malalignment between the patella and the trochlear groove of the femur, leading to improper patellar tracking (13). Consequently, the contact pressure on the lateral facet of the patella increases, with force being concentrated on the lateral aspect of the patella, thus elevating the stress in this region (13, 14). A finite element analysis demonstrated that a 10° increase in KER elevates average patellar cartilage stress by 11% in pain-free females (15). Additionally, KER has been identified as a key predictor of patellar cartilage stress during running in both pain-free individuals and those with PFP (16). Collectively, findings from these studies (11–16) suggest that KER during movements may increase PFJ stress, potentially contributing to a reduction in patellar cartilage thickness.

A key limitation of previous studies is that their findings were based on cadaveric specimens or computational models that simulate human movements and PFJ geometry (11–13). As a result, the relationship between cartilage morphology and KER in living participants remains unclear. Furthermore, while some modeling studies have incorporated subject-specific movements and cartilage geometry (15, 16), they did not evaluate whether the medial or lateral cartilage was more affected. This gap makes it uncertain which regional cartilage morphology is more strongly associated with KER. Lastly, the limited number of studies involving human participants further complicates our understanding of how cartilage morphology relates to KER during tasks that commonly exacerbate PFP, such as squatting, particularly across diverse populations.

To date, no studies have been conducted to examine how KER during weight-bearing activities relates to patellofemoral cartilage in persons with PFP, despite previous studies suggesting a potential link between KER and cartilage morphology in this population. Thus, the aim of this study was to investigate the association between patellar cartilage thickness and lower extremity kinematics, particularly KER during bilateral squatting, in individuals with and without PFP. Bilateral squatting was chosen for this study due to its functional relevance and better tolerance in individuals with PFP (1, 2). We hypothesized that thinner patellar cartilage would be associated with increased KER during bilateral squatting in individuals with and without PFP, suggesting a potential link between patellar cartilage morphology and knee frontal plane kinematics.

## 2 Methods

### 2.1 Participants

This is a cross-sectional study involving a post-analysis of data from 20 young female participants (10 PFP and 10 pain-free controls) previously enrolled in an earlier study (8). There were no significant differences in age, height, weight, and activity level between groups (Table 1). Participants in the PFP group were eligible if they experienced a gradual onset of retropatellar pain for at least 3 months (8). Informed consent was obtained from all participants, in accordance with the guidelines of the Health Sciences Institutional Review Board at the University of Southern California.

**Table 1:** Participants characteristics (mean ± standard deviation).

Characteristics	Patellofemoral pain group (*n* = 10)	Control group (*n* = 10)	*P*-value
Age (years)	25.1 ± 4.7	25.8 ± 6.1	0.78
Height (m)	1.77 ± 0.1	1.77 ± 0.1	0.97
Weight (kg)	59.7 ± 9.3	59.8 ± 7.1	0.45
Activity level (MET.min/week)	2,166.0 ± 969.1	1,944.0 ± 859.0	0.59

For participants in the PFP group, a physical examination was conducted to ensure other sources of pain were excluded (8). This evaluation included palpating the soft tissues surrounding the PFJ to accurately localize the pain. Participants with PFP were excluded if their pain originated from areas such as the quadriceps tendon, patellar tendon, patellar bursa, fat pad, menisci, or tibiofemoral joint (8). Additional exclusion criteria for participants in the PFP group included prior knee surgery, history of traumatic patellar dislocation, or the presence of implanted devices that could interfere with the magnetic field of the magnetic resonance imaging (MRI) (8).

The control group was matched to the PFP group in terms of age, height, weight, and activity levels (within 10% difference). Physical activity was assessed using the World Health Organization's Global Physical Activity Questionnaire (17). Control participants met the same selection criteria as the PFP group, except they had no history of PFP.

### 2.2 Procedures

Data collection occurred in two phases: MRI and biomechanical testing. In the PFP group, MRI scans were obtained from the affected limb; if both knees were symptomatic, the more painful side was selected. For control participants, the limb selected for MRI was matched to the corresponding side of their PFP counterpart. During biomechanical testing, data were collected from both limbs in all participants. However, for the PFP group, only data from the painful or more painful limb were analyzed. In the control group, analysis was conducted on the matched limb. This is to ensure consistency in assessing cartilage morphology and kinematics within the same limb for all participants.

#### 2.2.1 MRI assessment

To obtain patellar cartilage thickness, all participants were seated in a wheelchair with their legs raised to relieve pressure from the knee joint for at least 60 min prior to imaging. This procedure was used to prevent any external compression that might influence cartilage deformation (6, 18). All participants with PFP and pain-free controls underwent an MRI scan using a 3.0 Tesla General Electric scanner (GE Healthcare, Milwaukee, WI, USA). The patellar cartilage was assessed using axial plane images of the PFJ, acquired with a 3-dimensional (3D) fast Spoiled Gradient Recalled Echo (SPGR) sequence (TR = 16.3 ms, TE = 2.8 ms, flip angle = 10°, matrix = 384 × 160, field of view = 160 × 160 mm, slice thickness = 2 mm, knee positioned at 0° flexion, total scan time = 2 min).

#### 2.2.2 Biomechanical testing

During biomechanical testing, we aimed to quantify lower extremity kinematics during a bilateral squat. This task was selected because squatting is a common movement that often elicits pain in individuals with PFP (1, 2). Kinematic data were collected using an 11-camera Qualisys motion analysis system operating at 60 Hz (Qualisys Inc., Gothenburg, Sweden).

Before the testing, reflective markers were applied to all participants by the same investigator, a physical therapist. The anatomical markers were positioned on key bony landmarks, including the 1st and 5th metatarsal heads, medial and lateral malleoli, medial and lateral femoral epicondyles, the L5-S1 joint space, and bilaterally on the greater trochanters, and iliac crests, anterior superior iliac spines (ASISs). Rigid quadrilateral marker clusters were attached bilaterally on the lateral thigh and lower leg. Additionally, triads of rigid markers were placed on the heel counters of the shoes. After the markers were applied, a standing calibration trial was conducted to establish segmental coordinate systems and joint axes. Following this calibration, all anatomical markers were removed except for those at the iliac crests and the L5-S1 junction. The tracking clusters remained in place throughout the data collection session (19).

Following a standing calibration trial, participants were instructed to perform a bilateral squat, maintaining 45° of knee flexion. Participants were asked to maintain an upright posture for 10 s, holding their arms extended forward and keeping fingertip contact with a pole (8).

### 2.3 Data analysis

#### 2.3.1 Cartilage thickness

**Figure 1:**
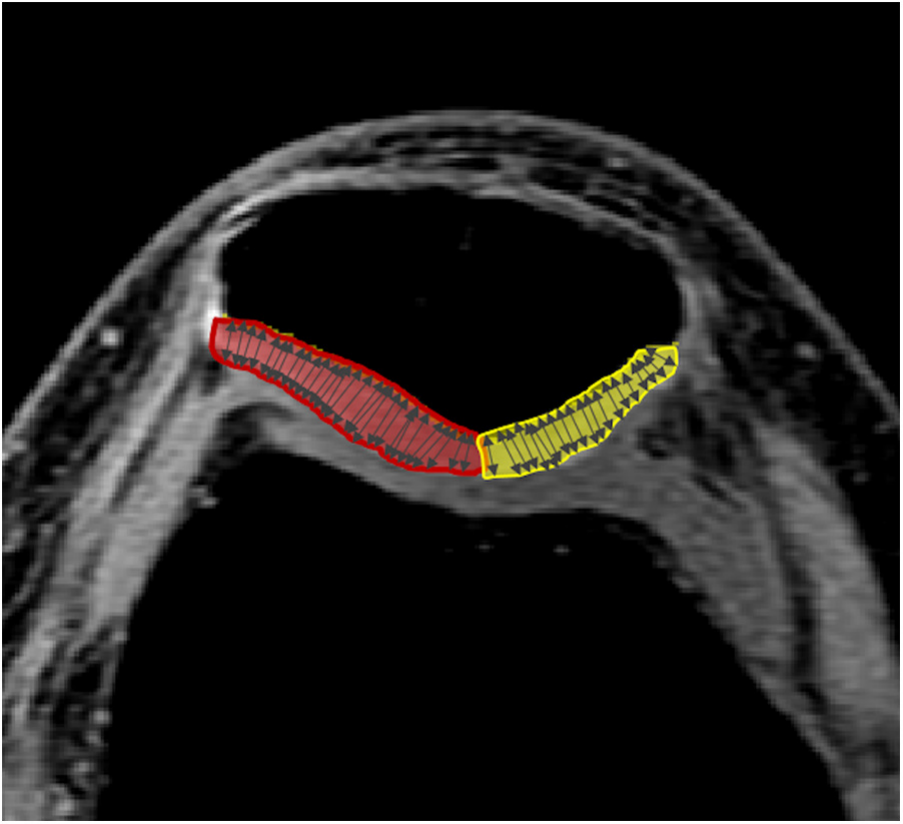
Quantification of patellar cartilage thickness. The red and yellow regions highlight the segmented lateral and medial patellar cartilage, respectively. Arrows indicate the measurements of the perpendicular distances between opposing voxels that define the boundaries of the patellar cartilage.

The medial and lateral patellar cartilage was manually segmented by a trained investigator using commercial software (sliceOmatic, Tomovision, Montreal, Québec, Canada) (Figure 1). Cartilage thickness was calculated by measuring the perpendicular distance between opposing voxels that defined the boundaries of the patellar cartilage, using a custom MATLAB program (MathWorks, Natick, Massachusetts). The medial and lateral patellar cartilage thicknesses were determined as the average distance within each region of interest. Total cartilage thickness was calculated as the mean of the measurements from both regions (6).

We selected three regions of the patellar cartilage—medial, lateral, and total—due to their distinct anatomical and mechanical roles. Individuals with PFP often exhibit patellar cartilage thinning, particularly on the lateral facet (6, 8). Given our focus on the relationship between KER and patellar cartilage, assessing region-specific cartilage morphology is essential. Additionally, total cartilage thickness serves as a comprehensive indicator of overall patellar cartilage health.

#### 2.3.2 Lower extremity kinematics

Reflective markers were labeled and digitized using Qualysis software (Qualisys Inc., Gothenburg, Sweden). Kinematic data for the hip, knee, and ankle in the sagittal, frontal, and transverse planes were quantified using Visual3D software (C-Motion, Rockville, MD). The kinematic data were filtered with a low-pass Butterworth filter (cutoff frequency: 6 Hz). Peak values of hip flexion, internal rotation, and adduction; knee external rotation and abduction; and ankle dorsiflexion, pronation, and external rotation during squatting were extracted for analysis.

### 2.4 Statistical analysis

All statistical analyses were conducted using IBM SPSS Statistics (version 28; IBM Corp., Armonk, NY). The Shapiro–Wilk test was used to assess the normality of the kinematic data and cartilage thickness. Except for the peak ankle inversion angle, which was not normally distributed, all variables showed normal distributions. Therefore, a non-parametric Spearman's rank correlation was used to assess the relationship between cartilage thickness and peak ankle inversion, while Pearson's correlation coefficient was applied for all other correlations between cartilage thickness and kinematic variables. Correlations were analyzed separately for participants with PFP, pain-free participants, and the combined group. Correlation coefficients were categorized as follows: small (0.1 < *r* ≤ 0.3), moderate (0.3 < *r* ≤ 0.5), large (0.5 < *r* ≤ 0.7), very large (0.7 < *r* ≤ 0.9), and extremely large (*r* > 0.9) (20). *T*-tests or Mann–Whitney *U*-tests were used to compare kinematic variables and cartilage morphology between groups, depending on the normality of the data distribution. Statistical significance was set at *p* < 0.05 for all analyses.

## 3 Results

**Figure 2:**
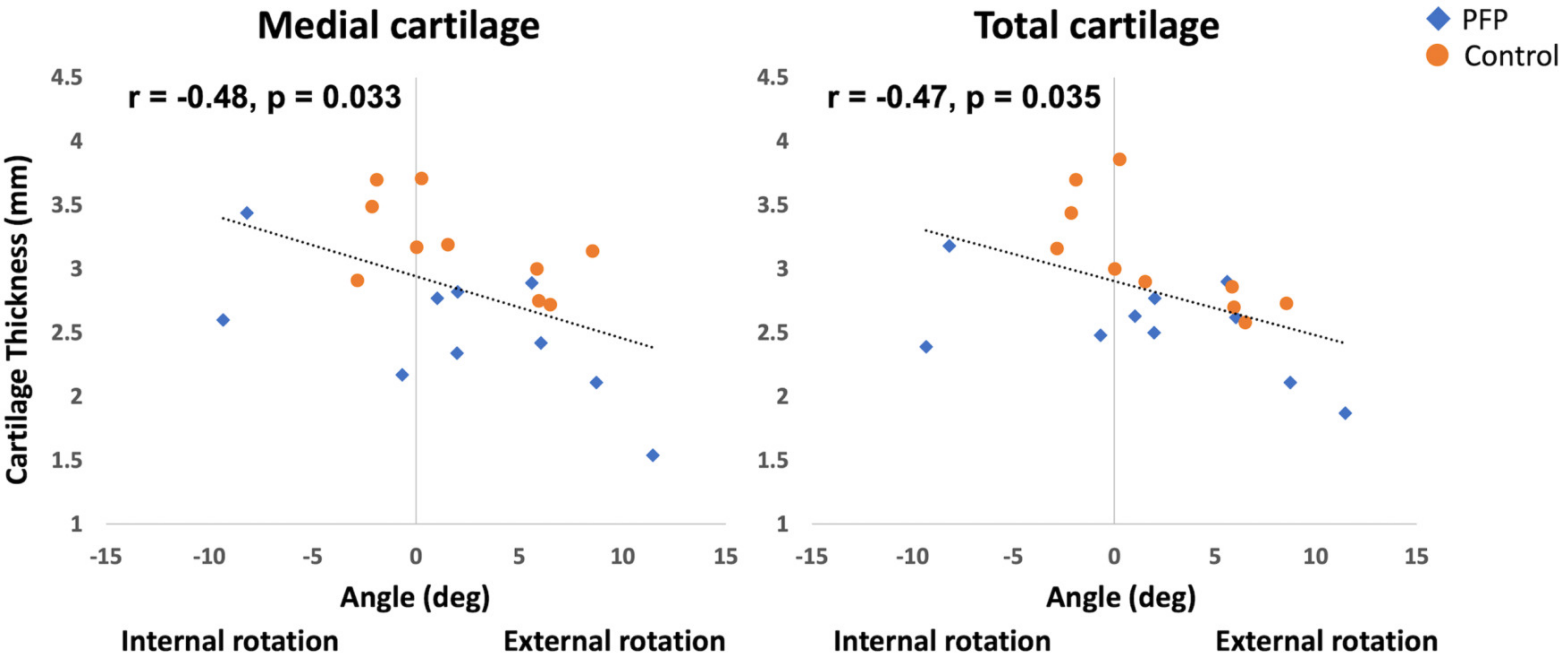
Correlations between medial and total patellar cartilage thickness and knee external rotation (KER) in participants with patellofemoral pain (PFP) and pain-free controls.

Statistically significant differences between the two groups were found only in cartilage thickness, while lower extremity kinematics showed no significant differences (Table 2). Across all participants, there was a significant moderate negative correlation between KER and both medial and total cartilage thickness (Figure 2). In individuals with PFP, the medial cartilage thickness showed a significantly large negative correlation with KER (*r* = −0.66 *p* = 0.037). In the control group, significant very large negative correlations existed between the total cartilage thickness and KER (*r* = −0.75 *p* = 0.013), and between the lateral cartilage thickness and KER (*r* = −0.79 *p* = 0.007). In addition, the medial and total cartilage thicknesses showed significant, very large (*ρ* = −0.70, *p* = 0.025) and large (*ρ* = −0.67, *p* = 0.033) negative correlations with ankle inversion, respectively.

**Table 2:** Comparisons of lower extremity kinematics and patellar cartilage thickness between groups.

Variables		Mean ± SD or Median (IQR)		*P*-value
		Patellofemoral pain group (*n* = 10)	Control group (*n* = 10)	
Lower extremity kinematics (deg)	Hip flexion	37.42 ± 10.56	36.46 ± 15.46	0.872^a^
	Hip internal rotation	−4.24 ± 4.26	−6.23 ± 2.17	0.204^a^
	Hip abduction	2.74 ± 3.68	3.43 ± 3.84	0.872^a^
	Knee flexion	50.52 (12.32–56.17)	52.63 (45.63–56.17)	0.143^b^
	Knee external rotation	1.87 ± 6.70	2.18 ± 4.16	0.902^a^
	Knee abduction	5.29 ± 3.74	5.65 ± 2.50	0.802^a^
	Ankle dorse flexion	25.13 ± 4.98	25.01 ± 5.23	0.960^a^
	Ankle inversion	4.92 ± 2.78	6.34 ± 2.27	0.228^a^
	Ankle external rotation	7.23 (−1.47–19.31)	5.79 (1.05–15.71)	0.465^b^
Cartilage thickness (cm)	Medial	2.51 ± 0.52	3.18 ± 0.36	0.004^a,^*
	Lateral	2.58 ± 0.31	3.01 ± 0.58	0.028^a,^*
	Total	2.55 ± 0.38	3.09 ± 0.44	0.008^a,^*

SD, standard deviation; IQR, interquartile range.

^a^ *t*-test.

^b^ Mann–Whitney *U*-test.

* Denotes a significant difference (*p* < 0.05).

No statistically significant correlations were observed between patellar cartilage thickness and hip movements in the sagittal, transverse, and frontal planes, as well as knee frontal plane movement and ankle movements in the sagittal and transverse planes, in the PFP, control, or combined groups (*p* > 0.05).

## 4 Discussion

### 4.1 Overall associations between patellar cartilage thickness and KER

To the authors’ knowledge, this is the first study to explore the relationship between patellar cartilage thickness and KER in individuals with PFP. We aimed to investigate the relationship between the cartilage thickness of the PFJ and lower limb kinematics in individuals with and without PFP. Consistent with our hypothesis, the findings indicated that decreased medial and total cartilage thicknesses were significantly associated with greater KER across all participants. Specifically, in individuals with PFP, thinner medial cartilage was linked to increased KER, while in the control group, thinner lateral and total cartilage thicknesses showed significant associations with greater KER. These results suggest that regardless of the existence of PFP, thinner patellar cartilage thickness significantly correlates with larger KER, known as a factor increasing stress on the PFJ (9, 10, 12, 16). Additionally, previous studies (6, 8) have reported that patellar cartilage thickness is significantly thinner in persons with PFP compared to those without. One possible explanation for this patellar cartilage morphological change is the cumulative effect of chronic, abnormal kinematics observed during movement in individuals with PFP (21–23). However, as our study, along with most previous research on cartilage thickness and kinematics in individuals with and without PFP (6, 16), used a cross-sectional design, we were unable to establish causality. Future long-term prospective studies are needed to determine whether greater KER leads to thinner patellar cartilage in individuals with PFP.

### 4.2 Associations between patellar cartilage thickness and KER in all participants

We found that thinner medial and total cartilage thickness was significantly related to larger KER in all participants with and without PFP. It has been suggested that mechanical stress is a key contributor to the deformation of the PFJ cartilage (24–26). Mechanical stress on the PFJ significantly increases as KER becomes higher in a cadaveric study (11) and in weight-bearing activities in human subject studies (15, 16). Increased KER is believed to elevate the stress on the PFJ due to the lateral tracking of the patella (12, 13). This heightened stress may lead to chronic excessive deformation of the patellar cartilage, ultimately resulting in cartilage thinning (12, 27). We believe that the association between thinner cartilage and greater KER may be due to excessive cartilage deformation from increased stress on the cartilage caused by higher KER. However, as this study is cross-sectional, the causal relationship remains unclear.

Previous studies have reported increased cartilage stress on the lateral facet of the patella with KER (12–14), a finding consistent with our observations in pain-free participants. However, our study found no significant relationship between lateral patellar cartilage thickness and KER in either the entire cohort or specifically in individuals with PFP. Despite this, a trend toward significance was observed (correlation coefficient = 0.394, *p* = 0.085) between lateral patellar cartilage thickness and KER in all participants with and without PFP. This lack of statistical significance may be attributed to the limited range and reduced variability of lateral cartilage thickness in individuals with PFP, potentially reducing the likelihood of detecting a statistically significant correlation. Specifically, we observed an overall thinner lateral cartilage with a smaller standard deviation in the PFP group (mean thickness: PFP = 2.58 ± 0.31 mm; control = 3.18 ± 0.61 mm). To investigate this further, we conducted Levene's test to assess differences in the variance of lateral cartilage thickness between the PFP group and the control group. The test revealed a significant difference in variance (Levene's Statistic = 6.74, *p* = 0.018) between groups, indicating that the PFP group had a smaller variability in the lateral patellar cartilage. These findings suggest that different variability in lateral cartilage thickness between groups may contribute to the observed relationship between lateral cartilage thickness and KER in our study. Therefore, larger-scale studies involving participants with and without PFP and a wider range of PFJ cartilage morphology are needed to examine the associations between patellar cartilage thickness and lower extremity kinematics in greater detail.

### 4.3 Associations between patellar cartilage thickness and KER in PFP participants

In the PFP group, our study found that thinner medial cartilage was associated with increased KER during bilateral squatting. Theoretically, although stress on the lateral cartilage of the PFJ increases with greater KER (12–14), KER is the strongest predictor of stress across the entire joint cartilage (16). This suggests that greater KER may similarly heighten stress on the medial cartilage, potentially contributing to our observed association between thinner medial cartilage thickness and KER in persons with PFP. Studies highlight that taping interventions aimed at reducing KER significantly decrease both the amount of KER during running (28) and pain (28, 29) in individuals with PFP. These findings suggest a potential link between KER and PFP through its influence on cartilage stress distribution. However, the limited body of causal research on the relationship between medial cartilage stress and KER in PFP underscores the need for further investigation. Furthermore, our study did not find a statistically significant association between lateral or total patellar cartilage thickness and KER during squatting in individuals with PFP. We believe this may be due to the limited range and reduced variability of lateral cartilage thickness in individuals with PFP, as previously discussed.

### 4.4 Associations between patellar cartilage thickness and KER in pain-free controls

In the control group, consistent with our hypothesis, thinner lateral patellar cartilage was significantly related to greater KER. This suggests that pain-free individuals with greater KER can have thinner lateral cartilage in the PFJ. In addition, thinner lateral patellar cartilage was also significantly related to greater ankle inversion in pain-free participants. Greater ankle inversion has been reported to be associated with increased KER, a factor that raises mechanical stress on the lateral cartilage of the PFJ, in individuals with PFP during gait (30–32). Thus, larger ankle inversion may be linked to thinner lateral cartilage due to this kinematic chain relationship between ankle inversion and KER. On the other side, Reischl et al. (33) have suggested that this relationship between KER and ankle inversion can vary among individuals (33–35). Additional analysis using Spearman's correlation coefficient revealed no significant negative relationship between KER and ankle inversion in the control group (*ρ* = −0.60, *p* = 0.067). Examination of the descriptive statistics revealed that ankle inversion in the control group exhibited a non-normal distribution, with one potential outlier showing an unusually large ankle inversion angle that likely skewed the correlation coefficient. We believe this distributional characteristic might contribute to the observed significant associations between patellar cartilage thickness and ankle inversion. Due to the limited sample size in this study, further research is needed to better understand these relationships.

### 4.5 Limitations

This study has several limitations. First, as a secondary analysis, we were unable to confirm an ideal sample size, which may have influenced the observed significant relationship between cartilage thickness and KER. Additionally, due to the cross-sectional design and the use of simple correlation analysis, the causal relationship between cartilage thickness and KER remains unknown. In this study, efforts were made to control for potential confounding factors between individuals with and without PFP by including only female participants and matching them for age, weight, height, and activity level. However, as other potential confounding variables influencing KER or patellar cartilage were uncertain, they were not controlled for. Future research should investigate additional confounding factors that may influence outcome measures between the PFP and control groups. Finally, while none of the participants with PFP reported pain during the squatting task, it is important to note that any abnormal kinematics on the painful side may have been compensated for by the asymptomatic limb. Future studies are needed to assess the associations between single-limb weight-bearing activities and PFJ cartilage morphology in persons with and without PFP.

## 5 Conclusion

This study is, to our knowledge, the first to investigate the relationship between patellar cartilage thickness and KER during bilateral squatting in individuals with and without PFP. Our data showed that thinner medial and total patellar cartilage was associated with increased KER during bilateral squatting in persons with and without PFP. In the PFP group, thinner medial patellar cartilage was associated with increased KER in individuals with PFP. In pain-free controls, thinner lateral and total patellar cartilage was associated with increased KER. These results suggest that increased KER during squatting may negatively affect patellar cartilage thickness, regardless of the presence of PFP. Therefore, clinicians should carefully assess KER during squatting mechanics when evaluating patients with or at risk of PFP, as abnormal movement patterns may serve as a contributor of altered PFJ cartilage morphology. Future research should investigate the influence of lower extremity kinematics, including KER during single-limb activities, on PFJ cartilage morphology to enhance clinical applications.

## Data Availability

The raw data supporting the conclusions of this article will be made available by the authors, without undue reservation.
